# Low Levels of Serum Tryptophan Underlie Skeletal Muscle Atrophy

**DOI:** 10.3390/nu12040978

**Published:** 2020-04-01

**Authors:** Soranobu Ninomiya, Nobuhiko Nakamura, Hiroshi Nakamura, Taku Mizutani, Yuto Kaneda, Kimihiro Yamaguchi, Takuro Matsumoto, Junichi Kitagawa, Nobuhiro Kanemura, Makoto Shiraki, Takeshi Hara, Masahito Shimizu, Hisashi Tsurumi

**Affiliations:** 1 First Department of Internal Medicine, Gifu University Graduate School of Medicine, Gifu 5011194, Japan; xenon2112@gmail.com (N.N.); ayapokopoko2@gmail.com (H.N.); taku.mizutani0912@gmail.com (T.M.); yuto-hayashi@outlook.com (Y.K.); chris_kimihiro@yahoo.co.jp (K.Y.); jkitagawa1128@gmail.com (J.K.); nkane@orion.ocn.ne.jp (N.K.); mshiraki-gif@umin.ac.jp (M.S.); shimim-gif@umin.ac.jp (M.S.); htsuru@gifu-u.ac.jp (H.T.); 2 Department of Hematology, Matsunami General Hospital, Gifu 5016062, Japan; haratake@muh.biglobe.ne.jp

**Keywords:** tryptophan, sarcopenia, cancer, indoleamine 2,3-dioxygenase, glycolysis

## Abstract

Sarcopenia is a poor prognosis factor in some cancer patients, but little is known about the mechanisms by which malignant tumors cause skeletal muscle atrophy. Tryptophan metabolism mediated by indoleamine 2,3-dioxygenase is one of the most important amino acid changes associated with cancer progression. Herein, we demonstrate the relationship between skeletal muscles and low levels of tryptophan. A positive correlation was observed between the volume of skeletal muscles and serum tryptophan levels in patients with diffuse large B-cell lymphoma. Low levels of tryptophan reduced C2C12 myoblast cell proliferation and differentiation. Fiber diameters in the tibialis anterior of C57BL/6 mice fed a tryptophan-deficient diet were smaller than those in mice fed a standard diet. Metabolomics analysis revealed that tryptophan-deficient diet downregulated glycolysis in the gastrocnemius and upregulated the concentrations of amino acids associated with the tricarboxylic acid cycle. The weights and muscle fiber diameters of mice fed the tryptophan-deficient diet recovered after switching to the standard diet. Our data showed a critical role for tryptophan in regulating skeletal muscle mass. Thus, the tryptophan metabolism pathway may be a promising target for preventing or treating skeletal muscle atrophies.

## 1. Introduction

Skeletal muscle atrophy and loss of strength is frequently observed and correlated with poor outcomes in patients with cancer [1]. We previously reported that low volume of skeletal muscle at diagnosis is associated with worse survival in patients with hematological malignancies [2,3]. Recently, metabolomics analysis has been used to understand how amino acid profiles are affected by glucose metabolism [4], aging [5], and cancer [6]. Toyoshima et al. [7] have reported an association between plasma amino acid profiles and age-related sarcopenia, and demonstrated that sarcopenia was correlated with lower tryptophan (Trp) and histidine (His) and higher glutamine (Gln) and proline (Pro) concentrations. Furthermore, plasma Trp and His concentrations are often reduced in patients with particular types of cancer [8]. These findings suggest that Trp may be a key essential amino acid associated with cancer-related sarcopenia; hence, analysis of serum Trp levels may help improve our understanding of the mechanisms underlying sarcopenia in patients with cancer.

Trp is involved in two important metabolic pathways, namely, kynurenine and serotonin synthesis pathways [9]. Most of Trp degradation occurs through the kynurenine pathway. Indoleamine 2,3-dioxygenase (IDO) is involved in the first and rate-limiting step of Trp metabolism, which produces kynurenine and several biologically active secondary metabolites, including kynurenic acid, anthranilic acid, and 3-hydroxyanthranilic acid [10]. These Trp metabolites block antigen-specific T-cell proliferation and induce T-cell death [11]. Since IDO is induced by inflammatory cytokines, such as interferon-γ, IDO expression represents an endogenous feedback mechanism that controls excessive immune responses [12]. IDO is also produced by cancer cells; therefore, Trp metabolism helps tumors evade the host immune responses and provides survival advantages to tumors [11]. Many studies have shown that a high level of IDO expression in tumors is a poor prognostic factor [13,14], and it is thought that IDO expression induces Trp deficiency in cancer patients.

In this study, we aimed to investigate the relationship between serum levels of Trp and skeletal muscles in the patients with diffuse large B-cell lymphoma. We also examined the effects of low levels of Trp on myoblast cell proliferation and differentiation, as well as on the energy and amino acid metabolism in murine skeletal muscles.

## 2. Materials and Methods

### 2.1. Patients

Fifty-four patients with primary diffuse large B-cell lymphoma, who were treated initially at the Gifu University Hospital from January 2012 to December 2013, were enrolled in this study. Patients’ serum samples were taken, and their clinical characteristics were recorded on admission, before any treatment started. The patients were not on any special diet. All patients received R-CHOP or R-THP-COP (rituximab plus, cyclophosphamide, pirarubicin, vincristine, and prednisone) chemotherapy. Each regimen included rituximab (375 mg/m^2^ on day 1), cyclophosphamide (750 mg/m^2^ on day 3), doxorubicin (DOX), or pirarubicin (THP) (50 mg/m^2^, administered as a 30-min infusion on day 3), vincristine (1.4 mg/m^2^ on day 3, with a maximal dose of 2.0 mg), and prednisolone (100 mg/day on days 3–7). R-CHOP and R-THP-COP chemotherapy cycles were repeated at 21-day intervals. Based on the physician’s advice, some patients received consolidative radiation after chemotherapy. The dose of radiation was 40 Gy in 20 fractions of 2 Gy administered for five days per week. Informed consent was obtained from all the patients regarding the use of the samples in this study. The study protocol was approved by the institutional review board.

### 2.2. Serum Tryptophan and Kynurenine Measurements

Serum Trp and kynurenine levels were measured by high-performance liquid chromatography with a spectrophotometric detector (Tosoh ultraviolet-8000, Tosoh, Tokyo, Japan) or fluorescence spectrometric detector (Hitachi, Tokyo, Japan) as previously reported [15], using a reverse-phase column (Brave ODS; 3 μm, 150 mm × 4.6 mm; Alltech, IL, USA) and a mobile phase composed of 0.1 M sodium acetate, 0.1 M acetic acid, and 1% acetonitrile, at a flow rate of 0.75 mL/min. The fluorescence of Trp was measured at excitation and emission wavelengths of 270 and 360 nm, respectively. Kynurenine was monitored by ultraviolet absorption at 355 nm.

### 2.3. Muscle Mass Measurements

The skeletal muscle mass was determined in the patients using stored images of computed tomography (CT) scans, which were obtained before any treatment. The skeletal muscle area was assessed from a single axial slice at the third lumber (L3) level. Hounsfield unit-based analyses of the images were performed using the dedicated SliceOmatic software version 4.3 (TomoVision, Montreal, QC, Canada) [16]. The skeletal muscle mass included the psoas muscle, quadratus lumborum, transversus abdominis, external and internal oblique muscles, rectus abdominis, and erector spinae, and the value was normalized for stature to determine the L3 skeletal muscle index (SMI).

### 2.4. Materials and Cell Culture Conditions

Trp, kynurenine, and Leu were purchased from Sigma–Aldrich (St. Louis, MO, USA). Trp- and Leu-depleted Dulbecco’s modified Eagle’s medium (DMEM) was obtained from Oriental Yeast Co., Ltd. (Tokyo, Japan). The C2C12 mouse muscle myoblast cell line was obtained from the American Type Culture Collection (Rockville, MD, USA). Human skeletal muscle myoblasts (HSMMs) were obtained from Lonza (Walkersville, MD, USA). Cells were maintained in a standard phenol red-free DMEM with high glucose (Sigma–Aldrich), containing Trp (78 μM) and Leu (800 μM) and supplemented with fetal bovine serum (10%), penicillin (100 U/mL), and streptomycin (100 μg/mL), and were incubated at 37 °C in humidified air containing 5% CO_2_. The myostatin enzyme-linked immunosorbent assay kit was purchased from R&D Systems, Inc. (Minneapolis, MN, USA).

### 2.5. Cell Proliferation Assay

Cell proliferation was assessed using an XTT cell proliferation kit (Roche Diagnostics GmbH, Mannheim, Germany), according to the manufacturer’s instructions. To examine the effects of Trp and kynurenine on C2C12 cell proliferation, cells were seeded into 96-well plates (1 × 10^4^ cells/well) and cultured overnight. After cell adhesion, the cell culture medium was changed to include specific concentrations of Trp or kynurenine, and the cells were incubated for 72 h. All assays were performed in triplicate.

### 2.6. Cell Differentiation and Myotube Formation Assay

C2C12 cells were seeded into a 12-well plate and cultured overnight. After cell adhesion, the culture medium was exchanged for differentiation medium, comprising DMEM supplemented with 2% heat-inactivated horse serum, with or without kynurenine, Trp, and Leu. The Leu-depleted medium, supplemented with 2% horse serum, contained 4.2 µM Leu, as the concentration of Leu in the horse serum was 181.4 µM. After incubation for seven days, the cell morphology was evaluated microscopically. The numbers of myotubes were counted in five fields per sample, and the average length was determined for 50 myotubes. Myotubes were fixed in 4% paraformaldehyde in phosphate buffered saline (PBS) for 10 min, followed by permeabilization with 0.2% Triton X-100 in PBS for 15 min. Cells were stained with anti-myosin heavy chain (MHC) antibody conjugated DyLight 488 (Novus biologicals; Centennial, CO, USA) for 24 h. The cells were counterstained with 4′6-diamidino-2-phenylindole dihydrochloride (DAPI) in PBS for 10 min. Fluorescent images were analyzed by using a BIOREVO BZ-9000 fluorescence microscope (Keyence, Osaka, Japan). The fusion index represented the number of nuclei in multinucleated MHC-positive cells, divided by the total number of nuclei in a field.

### 2.7. Animal Experiments

Nine-week-old C57BL/6 female mice (Japan SLC, Inc., Shizuoka, Japan) were fed a standard pellet diet containing Trp or a Trp-deficient pellet diet (Oriental Yeast Co., Ltd., Tokyo, Japan) for 21 days. Food and water were provided ad libitum. All mice were weighed twice a week, and the volume of the diet consumed was measured based on the volume of the diet remaining in a feeder. After 21 days, the Trp-deficient diet was replaced with the standard diet. At the end of the study, the mice were anesthetized, and the blood, tibialis anterior, and gastrocnemius muscles were collected. All animal experiments were performed in accordance with the guidelines of the laboratory animal manual and were approved by the Animal Welfare Committee of Gifu University.

### 2.8. Metabolome Analysis

Frozen skeletal muscle (approximately 50 mg) was plunged into 50% acetonitrile in Milli-Q water (1500 µL), containing internal standards (H3304–1002; Human Metabolome Technologies, Inc., Tsuruoka, Japan) at 0 °C. The tissue was placed in a tissue homogenizer (Micro Smash MS100R; Tomy Digital Biology Co., Ltd., Tokyo, Japan) and homogenized three times at 1500 rpm for 120 s. Then, the homogenate was centrifuged at 4 °C, 2300× *g* for 5 min. Subsequently, 800 µL of the upper aqueous layer was filtered by centrifugation through a 5 kDa cutoff filter (Millipore) at 4 °C, 9100× *g* for 120 min to remove proteins. The filtrate was concentrated by centrifugation and resuspended in 50 µL of Milli-Q water for capillary electrophoresis–mass spectrometry (CE-MS) analysis. The metabolome measurements were carried out at Human Metabolome Technologies, Inc. (Tsuruoka, Japan), and the concentrations of 116 targeted metabolites, including 20 amino acids, were measured and analyzed.

### 2.9. Statistics

Data are expressed as the means ± standard deviation. Statistical significance of differences in mean values was assessed using one-way analysis of variance, followed by Sheffe’s *t*-test. The serum Trp concentrations are presented as medians and ranges. The Mann–Whitney *U*-test was used for group comparisons, and a chi-squared test was used to compare categorical variables. A value of *p* < 0.05 was considered statistically significant. All statistical analyses were performed using the JMP software, version 13 (SAS Institute, Cary, NC, USA).

## 3. Results

### 3.1. Associations between Serum Tryptophan and Kynurenine Levels and Skeletal Muscle Mass

**Figure 1:**
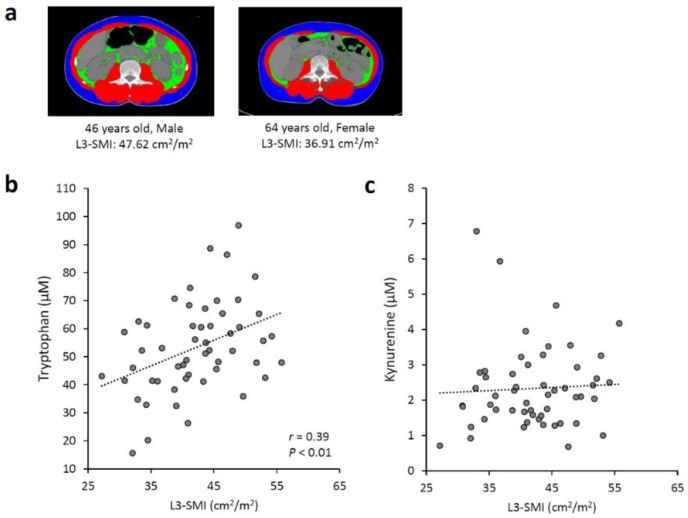
Associations between serum tryptophan and kynurenine levels and skeletal muscle mass. (**a**) Representative axial computed tomography images of the third lumbar (L3) vertebral region, with the skeletal muscle highlighted in red, of a male patient with a high L3-skeletal muscle index (SMI) (left) and a female patient with a low L3-SMI (right). Correlation between L3-SMI and (**b**) serum tryptophan and (**c**) kynurenine levels in 54 patients with diffuse large B-cell lymphoma. The Pearson product-moment correlation coefficient (*r* = 0.39) and *p* < 0.01 indicated a positive correlation between SMI and the serum Trp concentration.

We analyzed possible associations between the serum Trp and kynurenine concentrations and the skeletal muscle volume in the 54 patients with diffuse large B-cell lymphoma using CT images (Figure 1a). The Pearson product-moment correlation coefficient (*r* = 0.39) and *p* < 0.01 indicated a positive correlation between SMI and the serum Trp concentration (Figure 1b). There was no correlation between SMI and the serum kynurenine level (Figure 1c). Table 1 presents the patients’ baseline characteristics. The patients’ median Trp level was 58.17 µM (15.63–96.83 µM). Female sex and the presence of B symptoms were significantly associated with a low Trp level (*p* < 0.01, *p* < 0.05, respectively). Age > 60 years, poor performance status, elevated level of lactate dehydrogenase, multiple extranodal involvement, and advanced disease were not associated with a low Trp level. The median Trp level was significantly lower in the patients with L3-SMI < 41 cm^2^/m^2^ (42.64 µM (15.63–70.71 µM)) than in those with L3-SMI > 41 cm^2^/m^2^ (59.42 µM (35.85–96.83 µM)) (*p* < 0.01).

### 3.2. Effects of Low Levels of Tryptophan and Kynurenine on the Proliferation of and Myotube Formation by Skeletal Muscle Myoblast Cells

**Figure 2:**
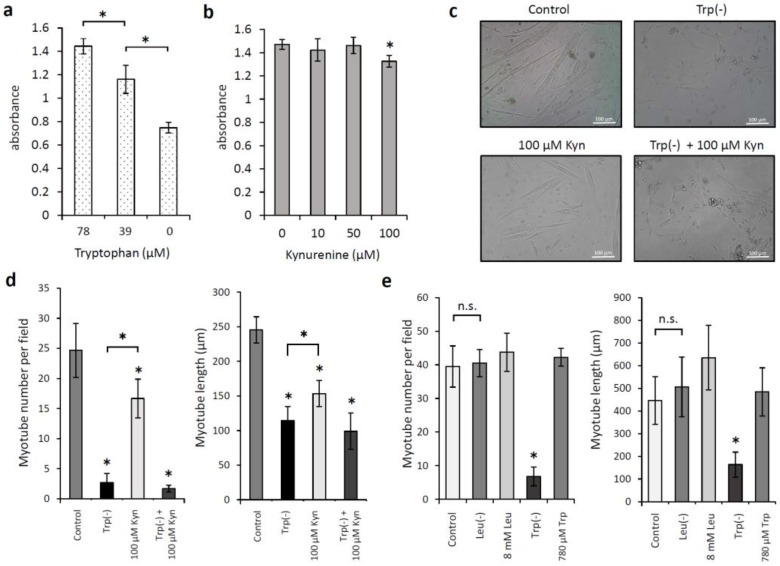
Effects of tryptophan (Trp), kynurenine (Kyn), and leucine (Leu) on C2C12 myoblasts. (**a**,**b**) Cell proliferation was determined after incubation of C2C12 myoblasts with the indicated concentrations of (**a**) Trp and (**b**) Kyn for 72 h. (**c**–**e**) Cell differentiation was determined based on the myotube formation by C2C12 myoblasts after incubation in differentiation medium (DMEM supplemented with 2% horse serum) under the indicated conditions for seven days. (**c**) Cell morphology. (**d**,**e**) Numbers of myotubes and the myotube length. * *p* < 0.05.

**Figure 3:**
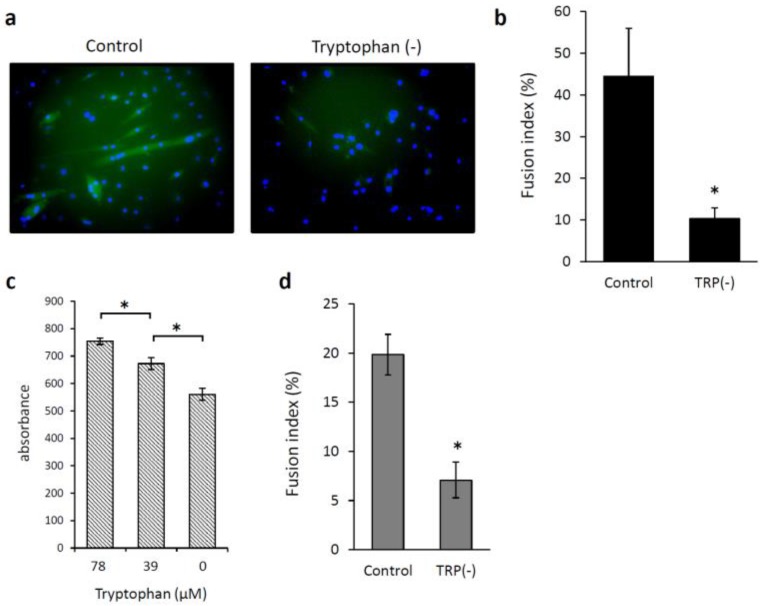
Tryptophan (Trp) depletion reduces the fusion indexes in C2C12 and human skeletal muscle myoblasts (HSMMs). (**a**) Immunofluorescence images of C2C12 cells cultured in differentiation medium with or without Trp for 5 days and labeled with DyLight 488-conjugated anti-myosin heavy chain (green) and DAPI (blue). (**b**) Total fusion index represents the number of nuclei in multinucleated myotubes, divided by the total number of nuclei in a field, with a myotube defined as a cell with at least two nuclei. (**c**) Proliferation and (**d**) fusion index of HSMMs. * *p* < 0.05.

We examined whether low levels of Trp or kynurenine inhibited C2C12 cell proliferation. When C2C12 myoblasts were incubated with Trp at 0, 39, and 78 μM for 72 h, the cell proliferation was significantly reduced in the absence of Trp and increased in a concentration-dependent manner upon the addition of 39 and 78 μM Trp (*p* < 0.05; Figure 2a). Kynurenine at concentrations <100 µM did not inhibit C2C12 cell proliferation (Figure 2b). We also examined the effects of Trp and kynurenine on C2C12 cell differentiation, specifically on myotube formation. The medium change from 10% fetal bovine serum to 2% horse serum induced myogenic differentiation in C2C12 cells. Figure 2c shows the morphology of the cells after they were cultured with 2% horse serum for seven days. Cell incubation with kynurenine (100 µM) without Trp significantly reduced the number and length of myotubes compared with those of cells incubated in standard DMEM (*p* < 0.05; Figure 2d). C2C12 cell differentiation significantly reduced in the absence of Trp compared with that observed under kynurenine addition (*p* < 0.05; Figure 2d). Next, we examined whether low levels of Leucine (Leu) would affect C2C12 cell differentiation. When cells were incubated in a Leu-depleted DMEM containing 2% horse serum, the number or length of myotubes did not change compared with those of cells incubated in a standard DMEM (Figure 2e). Cell incubation in a medium containing 8 mM Leu tended to increase the length of myotubes; however, the difference was not significant. C2C12 cell differentiation was restored when 780 µM Trp was added to the Trp-depleted DMEM. Myotubes were fluorescently labeled using an anti-MHC antibody and DAPI nuclear staining (Figure 3a), and the fusion index was calculated to assess myotube formation. Low levels of Trp significantly prevented the fusion of C2C12 myoblasts into myotubes (*p* < 0.05; Figure 3b). HSMMs proliferation and myotube formation were also reduced under low levels of Trp condition (Figure 3c,d).

### 3.3. Tryptophan-Deficient Diet Induces Skeletal Muscle Atrophy in Mice

**Figure 4:**
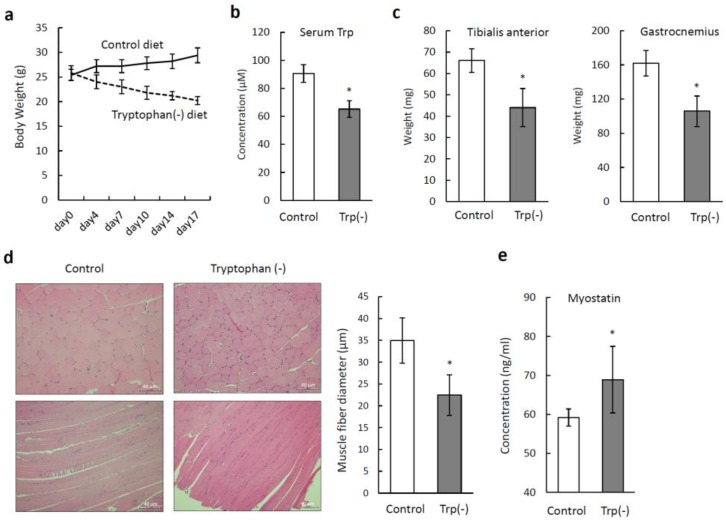
Effects of a tryptophan (Trp)-deficient diet on the body weight and skeletal muscles of mice. (**a**) Time courses of body weight changes, measured twice a week, in C57BL/6 mice (nine-week-old, female) fed a standard diet (*n* = 5) or a Trp-deficient diet (*n* = 5). (**b**) Serum Trp levels on day 21. (**c**) Weights of the tibialis anterior and gastrocnemius collected after 21 days. (**d**) Pathological changes in the tibialis anterior were evaluated by staining with hematoxylin and eosin, and cross-section muscle fiber diameters were measured. Scale bars = 40 µm. (**e**) Serum myostatin levels on day 21. *p < 0.05.

We examined the effects of a standard diet and a Trp-deficient diet on the skeletal muscles of mice. Figure 4a shows the changes in the body weight over time. At the beginning of the experiment, the body weights did not differ between the two groups. During the experiment, the mice fed the standard diet gradually gained weight, and those fed the Trp-deficient diet gradually lost weight. The volumes of the food consumed by the mice during the experiment did not differ between the groups. None of the mice fed the Trp-deficient diet died during the experimental period. The mean serum Trp level in the mice fed the Trp-deficient diet (65.2 ± 5.9 µM) was significantly lower than that in the mice fed the standard diet (90.6 ± 6.3 µM) on day 21 (*p* < 0.05; Figure 4b). The muscle weights of the tibialis anterior and gastrocnemius from the mice fed the Trp-deficient diet were significantly lower than those from the mice fed the standard diet (*p* < 0.05; Figure 4c). The mean fiber diameter in the tibialis anterior from the mice fed the Trp-deficient diet was significantly smaller (22.44 ± 4.65 µm) than that from the mice fed the standard diet (34.96 ± 5.18 µm) (*p* < 0.05; Figure 4d). We also measured the serum levels of myostatin. The mean serum myostatin level in the mice fed the Trp-deficient diet (68.89 ± 8.54 ng/mL) was significantly higher than that in the mice fed the standard diet (59.18 ± 2.19 ng/mL) (*p* < 0.05; Figure 4e). There were few centrally located nuclei in the skeletal muscles of both groups. There were no obvious abnormalities in the skeletal muscle structure, such as fat or collagen accumulation in the mice fed the Trp-deficient diet.

### 3.4. Tryptophan-Deficient Diet Changes the Amino Acid Profile in Skeletal Muscles

**Figure 5:**
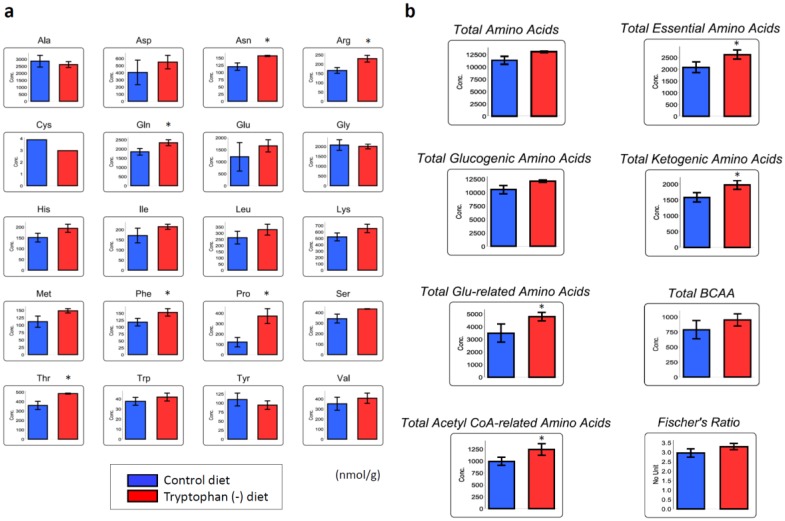
Differences in amino acid concentrations in the gastrocnemius skeletal muscles of mice fed a control diet and tryptophan (Trp)-deficient diet. (**a**) Concentrations of 20 amino acids. (**b**) Concentrations of the total amino acids, essential amino acids, glucogenic amino acids, ketogenic amino acids, glutamate (Glu)-related amino acids (Arg, Gln, Glu, His, and Pro), branched-chain amino acids (BCAAs), and acetyl coenzyme A-related amino acids (Ile, Leu, Lys, and Trp) and Fisher’s ratio (BCAAs/(Phe + Trp + Tyr)). Data are the means and standard deviations (nmol/g; n = 3). *p < 0.05.

The concentrations of Trp in the muscle of gastrocnemius did not differ between the mice fed the Trp-deficient and standard diets. Figure 5a shows amino acid concentrations in the gastrocnemius from both groups. The concentrations of asparagine (Asn), arginine (Arg), Gln, phenylalanine (Phe), Pro, and threonine (Thr) were significantly elevated in the gastrocnemius from the mice fed the Trp-deficient diet compared with those in the gastrocnemius from the mice fed the standard diet. The concentration of Pro in the gastrocnemius from the mice fed the Trp-deficient diet was approximately three times higher than that in the gastrocnemius from the mice fed the standard diet. None of the amino acid levels in the gastrocnemius from the mice fed the Trp-deficient diet were lower than those in the gastrocnemius from the mice fed the standard diet. The overall concentrations of the essential amino acids, ketogenic amino acids (isoleucine (Ile), Leu, lysine (Lys), phenylalanine, Thr, Trp, and tyrosine), glutamate (Glu)-related amino acids (Arg, Gln, Glu, His, and Pro), and acetyl coenzyme A-related amino acids (Ile, Leu, Lys, and Trp) in the gastrocnemius from the mice fed the Trp-deficient diet were significantly higher than those in the gastrocnemius from the mice fed the standard diet (Figure 5b). The concentrations of the branched-chain amino acids (BCAAs), namely, Ile, Leu, and valine (Val), did not differ between the groups.

### 3.5. Serum Tryptophan Deficiency Induces Metabolic Alterations in Skeletal Muscles

**Figure 6:**
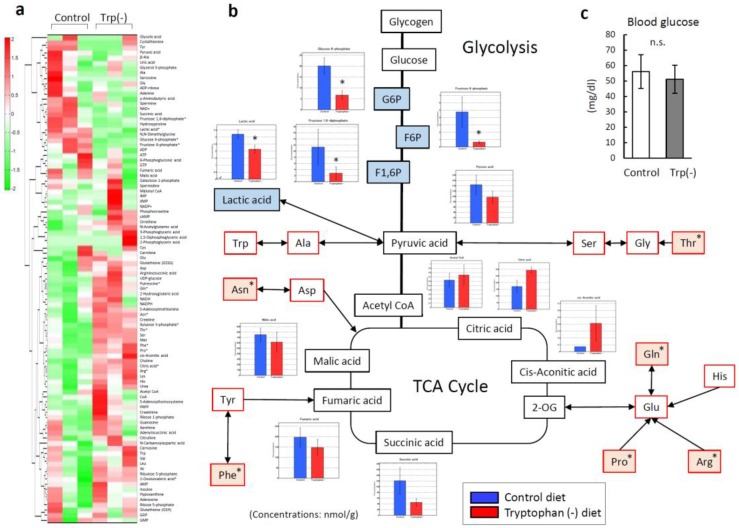
Metabolic responses in the skeletal muscle of mice fed a control diet and a tryptophan (Trp)-deficient diet. (**a**) Metabolic pathway enrichment analysis. (**b**) Levels of metabolites involved in the glycolytic pathway and tricarboxylic acid cycle in the gastrocnemius. (**c**) Blood glucose levels on day 21. Data are the means and standard deviations (n = 3). * *p* < 0.05. G6P: glucose 6-phosphate; F6P: fructose 6-phosphate; F1,6P: fructose 1,6-diphosphate.

To investigate why some amino acids increased in the skeletal muscle of the mice fed the Trp-deficient diet, metabolic changes were analyzed using targeted metabolomics and capillary electrophoresis–mass spectrometry (CE-MS). Figure 6a shows a heat map of 116 targeted metabolites in the gastrocnemius from both groups of mice. Compared with that from the mice fed the standard diet, the gastrocnemius from the mice fed the Trp-deficient diet contained significantly lower levels of intermediate metabolites of the glycolysis pathway, including glucose 6-phosphate (*p* = 0.01), fructose 6-phosphate (*p* = 0.02), and fructose 1,6-diphosphate (*p* = 0.04) (Figure 6b), similar levels of pyruvic acid, and elevated levels of Thr (*p* = 0.04). The gastrocnemius muscles from the two groups did not differ in terms of the levels of intermediate metabolites of the tricarboxylic acid (TCA) cycle or the levels of blood glucose (Figure 6c).

### 3.6. Recovery of Skeletal Muscle Atrophy Induced by a Tryptophan-Deficient Diet

**Figure 7:**
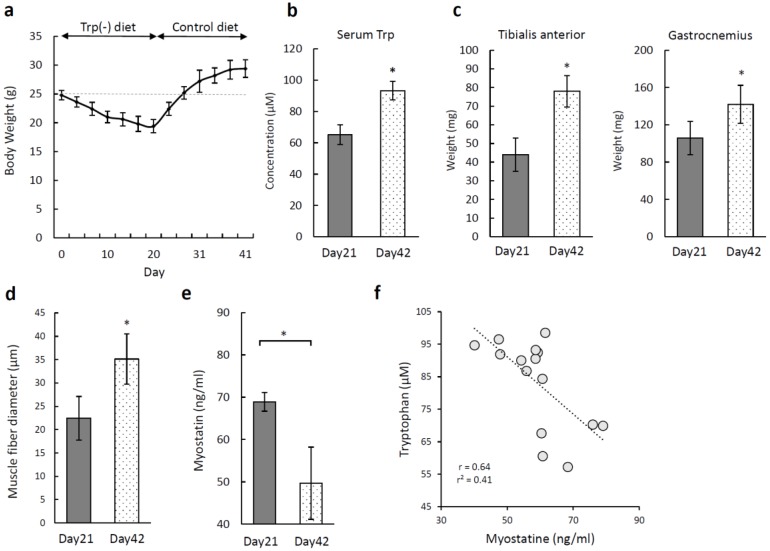
Recovery of the skeletal muscle mass in mice after replacement of a tryptophan (Trp)-deficient diet with a standard diet. (**a**) Body weight changes measured twice per week. Female C57BL/6 mice (nine-week-old) were fed a Trp-deficient diet for 21 days, followed by 21 days of a normal diet (n = 5). (**b**) Serum Trp levels on days 21 and 42. (**c**) Weights of the tibialis anterior and gastrocnemius on days 21 and 42. (**d**) Diameters of muscle fibers in the hematoxylin- and eosin-stained tibialis anterior. (**e**) Serum myostatin levels on days 21 and 42. (**f**) Correlation between serum tryptophan and myostatin levels in C57BL/6 mice (n = 15). The Pearson product-moment correlation coefficient (r = 0.64) and *p* < 0.01. * *p* < 0.05.

We fed the Trp-deficient diet to mice for 20 days, followed by the standard diet for a further 20 days, to examine whether the skeletal muscle atrophy would recover. Figure 7a shows the changes in the body weight over time. The mice fed the Trp-deficient diet for three weeks lost 6 g, but their weights recovered during the first week on the standard diet and continued to increase. The mean serum Trp level was significantly higher on day 42 (93.31 ± 2.49 µM) than on day 21 (*p* < 0.05; Figure 7b). The weights of the tibialis anterior and gastrocnemius also recovered (Figure 7c). The mean muscle fiber diameter in the tibialis anterior was higher on day 42 (35.12 ± 5.38 µm) than on day 21 (Figure 7d), while the mean serum myostatin level was significantly lower on day 42 (49.67 ± 7.08 ng/mL) than on day 21 (*p* < 0.05; Figure 7e). There was a negative correlation between the levels of serum Trp and serum myostatin (Figure 7f).

## 4. Discussion

This study showed a critical role for tryptophan in regulating skeletal muscle mass. Our results showed a positive correlation between serum Trp levels and skeletal muscle mass in the patients with diffuse large B-cell lymphoma. We also showed that tryptophan deficiency and refeeding induced reversible skeletal muscle loss in mice and that C2C12 myoblast proliferation and fusion were impaired with low tryptophan. Skeletal muscle atrophy is a significant predictor of mortality and morbidity in patients across a variety of diseases including cancer [3], but there are no clinical therapies approved to treat or prevent sarcopenia.

Trp is one of the most important amino acids related to cancer progression [11]. The overexpression of IDO, which is the first enzyme in the kynurenine pathway, has been described in cells from several types of cancers [17]. The kynurenine pathway is highly regulated in the immune system, where it promotes Trp breakdown in response to immune activation against cancer [11]. IDO expression in the tumor induces low levels of Trp and high levels of kynurenine in the plasma. In a clinical trial with an IDO inhibitor, the kynurenine/tryptophan ratio in the serum was decreased in patients receiving the IDO inhibitor [18]. A high level of IDO expression, which leads to Trp-associated metabolic abnormalities, is a significant risk factor for several cancers [18]. Low levels of Trp and high levels of Trp metabolites, including kynurenine, can inhibit the cytotoxicity of T cells and natural killer cells [19]; hence, IDO plays a major role in cancer immunodeficiency [20]. We found that lower levels of serum Trp were positively correlated with lower skeletal muscle volumes in patients with diffuse large B-cell lymphoma. In contrast, serum kynurenine level was not associated with skeletal muscle volume. Miyagi et al. [8] have compared plasma amino acid profile using a pooled data set including all cancer patients and controls. They reported alterations in amino acid profiles even in patients with early-stage cancer, most of whom had no obvious symptoms, such as the loss of appetite. The plasma concentrations of Gln, Trp, and His were significantly decreased in all of the cancers except pancreatic cancer, and none of the amino acids showed increased concentrations across all types of cancer. Serum levels of Trp in patients with cancer might be low from the point with early clinical stage because of overexpression of IDO in tumor. It is best to demonstrate that low serum Trp could cause skeletal muscle atrophy using tumor-bearing mice. We used Trp-deprived media and mice fed a tryptophan-deficient diet to investigate the effect of Trp deficiency on skeletal muscle. Our mouse model can only focus on the effects of decreased serum Trp on the skeletal muscle.

Cancer growth requires Glu, Gln, and Asp for purine and pyrimidine synthesis, and serine for membrane lipid component synthesis in addition to essential amino acids. Demand for certain amino acids may lead to a gradual loss of muscle mass, which causes the protein turnover in tissues. Leu is a key amino acid for the control of translation initiation in muscle cells [21]. Leu is the most effective stimulator of the mammalian target of rapamycin (mTOR) signaling pathway [22,23], but our results suggest that Leu is not essential for myotube formation in C2C12 cells. On the other hand, our data demonstrated that C2C12 myoblast proliferation and fusion were impaired with low tryptophan.

In skeletal muscle, the availability of amino acids, of Trp in particular, is essential for protein synthesis and normal protein turnover [24]. The absence of Trp in the diet had consequences both on embryo development, especially on regular sexual differentiation and skeletal muscle development, trophism, and contractility in rats [25]. In the present study, the weights of body and skeletal muscle of mice fed the Trp-deficient diet declined, even though the volumes of food eaten by the mice in the two groups were the same. The time course of the body weight change was similar to that described in an earlier publication [26], in which the authors suggested that a reduction in body motion, caused by a change in serotonin level, led to weight loss. Trp is one of the essential amino acids, which can be gained only from diet. However, the serum level of Trp remained at 65 µM in the mice fed the Trp-deficient diet for 20 days. Our findings suggest that the mice fed the Trp-deficient diet might have obtained serum Trp by catabolizing skeletal muscle proteins into amino acids.

Increased muscle protein degradation or decreased protein synthesis or both are critical factors in muscle atrophy. Degradation of muscle protein is mainly regulated by the ubiquitin–proteasome system. Overproduction of myostatin and activins, inflammatory responses, and impaired insulin-like growth factor 1 (IGF-1)-dependent protein synthesis are known to be closely related to the pathogenesis of muscle atrophy [27]. Myostatin is a critical negative regulator of skeletal muscle growth. Myostatin negatively regulates Akt pathway activity, which promotes protein synthesis, and increases ubiquitin–proteasome activity to induce atrophy through FOXO transcription factors [28]. Myostatin is an important regulator of the skeletal muscle because it negatively controls the proliferation of satellite cells, which are skeletal muscle-resident cells, and the differentiation of myoblasts into myotubes [29]. We found that the serum levels of myostatin were clearly elevated in the mice fed the Trp-deficient diet and significantly declined as the skeletal muscle recovered. Our data also indicated a negative correlation between serum levels of Trp and myostatin. Trp supplementation causes significant increases in the skeletal muscle levels of insulin-like growth factor-1, leptin, and follistatin, which is a myostatin antagonist [30].

Interestingly, the concentrations of Trp in the skeletal muscle did not drop even when the serum Trp concentrations declined in the mice fed the Trp-deficient diet. Meanwhile, the concentrations of some other amino acids rose in the gastrocnemius when the serum Trp concentration declined. When intracellular amino acid levels begin to fall, amino acid-sensing pathways reduce protein synthesis to maintain viable intracellular amino acid concentrations [21]. Recent evidence suggests that the mTOR complex 1 amino acid-sensing pathway [21] contributes to the reduction of protein synthesis and, ultimately, to atrophy of aged skeletal muscles [30]. Our analysis of the metabolome of skeletal muscles from the mice fed the Trp-deficient diet showed a clear reduction in glycolysis. Lee at al. demonstrated that glycolysis is linked to the mTOR complex 1 pathway via direct binding of glyceraldehyde-3-phosphate dehydrogenase (GAPDH) to Rheb [27]. Low glycolytic flux enhances the binding of GAPDH and Rheb, ultimately suppressing mTOR signaling. Thus, the GAPDH–Rheb axis is responsible for close crosstalk between the glycolytic and the mTOR pathways. The mTOR signaling pathway integrates a wide variety of extra- and intracellular signals, including insulin, nutrient availability, and cellular energy status to regulate protein synthesis. Reduction of glycolysis due to Trp deficiency may suppress mTOR signaling, which is a key regulator of protein synthesis in the skeletal muscle. Skeletal muscles from the mice fed the Trp-deficient diet showed increases in the concentrations of amino acids associated with the TCA cycle. These amino acids are metabolized to molecules involved in the TCA cycle, while Trp is a glycogenic amino acid, which is metabolized to pyruvic acid. These metabolic changes seem to compensate for the reduction in energy production from pyruvic acid in the skeletal muscle. Indeed, the Pro level was significantly elevated in the mice fed the Trp-deficient diet, which concurs with the data describing the serum amino acids associated with sarcopenia in older people [7]. Glucose is the main energy fuel for humans and is stored as glycogen, primarily in the liver and skeletal muscle. Liver glycogen maintains blood glucose levels, while skeletal muscle glycogen is utilized during high-intensity exertion [31]. Eleftheriadis et al. [32] reported that IDO reduced glycolysis and glutaminolysis by activating general control nonderepressible 2 kinase (GCN2K) in CD4^+^ T cells.

Benjamin et al. [33] have reported that dysfunction of skeletal muscle stem cells causes irreversible age-related muscle atrophy. We did not assess muscle satellite cells. However, the contribution of satellite cells to the development of sarcopenia is controversial [34]. Our data showed that the skeletal muscle atrophy caused by low levels of Trp was reversible in mice. Therefore, dietary Trp supplementation could also be effective for the improvement or prevention of skeletal muscle atrophy in age-related sarcopenia and muscular dystrophy patients whose serum Trp is decreased. Trp supplementation must be carefully managed in patients with cancer because it may promote tumor growth through increases in Trp metabolites, including kynurenine, which suppress the immune responses to tumors. Consequently, it may be preferable to suppress the function of IDO, expressed in cancer cells, and to allow the skeletal muscles to use Trp. Although a small molecule IDO1 inhibitor did not show promising results in a cancer immunotherapy phase III clinical trial [35], IDO inhibitors could impede skeletal muscle atrophy, which is caused by overexpression of IDO in tumors and mediated by Trp metabolism. Another point to consider is the risk for patients with liver cirrhosis to develop hepatic encephalopathy because Trp increases serotonin synthesis [36].

We did not examine skeletal muscles from tumor-bearing mice, which is a limitation of this study. Tumors secrete a variety of factors, while this study only focused on the effects of Trp on the skeletal muscle. This study was a retrospective analysis in a specific population of cancer, diffuse large B-cell lymphoma, which is another limitation. Further investigations targeting some other types of cancer will be needed to provide evidence for a link between Trp metabolism and cancer-related sarcopenia. The study to assess the time course relationship between skeletal muscle atrophy with serum Trp might show the usefulness of serum Trp for a valuable biomarker of sarcopenia development and progression. In this study, female patients with DLBCL had significantly lower serum tryptophan levels. However, a positive correlation was observed between SMI and serum Trp levels in both male and female patients. It could be interesting to examine if there is a sex-related difference in the effects of tryptophan deficiency, but only female mice were used in this study. Assessment of a single axial slice at the L3 level can provide an estimate of the total body skeletal muscle volume [37]. CT is generally performed before the treatment of cancer, and software is now commercially available; therefore, evaluation of skeletal muscles using CT images can be easily performed for cancer patients at any institution. On the other hand, a CT scan is not useful for measuring muscle quality, and there is not enough volume of CT scan data in healthy subjects to compare with cancer patients. We examined the tibialis anterior and gastrocnemius, which are Type II, fast-twitch muscles, in mouse experiments because these muscles better reflect skeletal muscle function such as gait. The walking speed also reflects the function of skeletal muscles and is used as a diagnostic criterion for sarcopenia [38].

In summary, our data showed, for the first time, that low levels of serum Trp underlie skeletal muscle atrophy in the patients with diffuse large B-cell lymphoma. The results from the present study also indicated that Trp-deficient diet reduced serum levels of Trp and induced reversible skeletal muscle loss in mice. Targeting the Trp metabolism, which is mediated by IDO, might be a promising strategy for preventing or treating sarcopenia, especially in cancer patients who overexpress IDO.

## Figures and Tables

**Table 1:** Clinical characteristics and serum tryptophan levels in the patients with diffuse large B-cell lymphoma.

Characteristic		No.	Serum Tryptophan (µ M)		
			Median	Range	*P*-Value
	All patients	54	58.17	15.63–96.83	
Sex	male	34	56.13	15.63–96.83	<0.01
	female	20	43.06	20.19–70.71	
Age (years)	<60	14	55.71	26.3–86.41	0.33
	≥60	40	51.16	15.63–96.83	
PS	0–1	42	52.09	15.63–86.41	0.28
	2–4	12	52.25	26.3–96.83	
LDH	normal	24	54.99	15.63–96.83	0.22
	increased	30	48.8	26.3–86.41	
Extranodal sites	0–1	40	51.62	15.63–86.41	0.30
	≥2	14	53.62	26.3–96.83	
CS	I/II	25	56.13	15.63–78.58	0.18
	III/IV	29	47.93	26.3–96.83	
B symptoms	Absent	36	56.13	20.19–96.83	<0.05
	Present	18	43.56	15.63–74.55	
L3-SMI (cm^2^/m^2^)	<41	27	42.64	15.63–70.71	<0.01
	≥41	27	59.42	35.85–96.83	

PS: performance status; LDH: lactase dehydrogenase; CS: clinical stage; L3-SMI: third lumbar skeletal muscle index.
